# Gene expression and highly diluted molecules

**DOI:** 10.3389/fphar.2014.00237

**Published:** 2014-11-12

**Authors:** Marta Marzotto, Debora Olioso, Paolo Bellavite

**Affiliations:** Section of General Pathology, Department of Pathology and Diagnostics, University of VeronaVerona, Italy

**Keywords:** *Gelsemium sempervirens*, high dilutions, gene expression regulation, microarray technology, neurocyte cell line, Homeopathy

Plants of the genus *Gelsemium* have measurable effects on anxiety-like symptoms in laboratory models (Magnani et al., 2010; Liu et al., 2013; Meyer et al., 2013; Jin et al., 2014). Searching for a possible mechanism of action, we found that very low doses and homeopathic dilutions of *Gelsemium sempervirens* (*Gelsemium*) modulate the expression of genes involved in neuronal functions (G-protein coupled receptor signaling pathways, calcium homeostasis, inflammatory response and receptors) (Marzotto et al., 2014; Olioso et al., 2014).

The commentary's first criticism is that “the search for an involvement of neural genes related to anxiety/depression or mood disorders is biased by the expression of human genes having no orthologs/homologs in mice, where the authors reported evidence about *Gelsemium* action on behavioral tests in animal anxiety models.” Frankly, we do not see what type of bias can be found in our line of research: *Gelsemium* is traditionally used as a remedy for anxiety-related symptoms in humans; we have shown that it works in mice models (Magnani et al., 2010; Bellavite et al., 2012), and we wanted to study its mechanism of action at the molecular level, using human neurocyte cell lines. This type of experimental procedure, which addresses various knowledge gaps using both animal studies and *in vitro* models, is very common in pharmacological studies.

Furthermore, the commentary (Chirumbolo, 2014) states that “some genes indicated to be downregulated by *Gelsemium* 2c, should not be expressed by neuronal cells (e.g., CD163, MPO, C8B, LST1, TREM2, notoriously expressed in immune cell).” Actually, it is well known that neurons express genes of cellular pathways also involved in cytokine/chemokine and immune responses. Genes related to immune and inflammatory responses are expressed in SH-SY5Y cells, as reported by many researchers (Gatta et al., 2011; Toyama et al., 2011; Cui et al., 2012; Xu et al., 2013).

Although *Gelsemium* contains several different compounds (Dutt et al., 2010; Jin et al., 2014), the major active alkaloid of this plant is gelsemine. In the cited commentary we read that “concentration of gelsemine was not assessed, as it was solely calculated on previous spectrometry investigations and new preparations, from ethanol draw extracts, were not further quantified by analytical chemistry.” This statement is misleading because the concentration of gelsemine which we correctly reported (0.021 g/100 ml, corresponding to 6.5 × 10^−4^ mol/L) (Marzotto et al., 2014, p. 2) was precisely determined by liquid chromatography (not “spectrometry”) in the mother tincture from which the samples were prepared. The concentration of UV-VIS absorbing substances decreased by a factor of 100 at each centesimal dilution step, to become analytically undetectable after a few passages, as we have shown by means of spectrometry in our paper. What we were interested in was to check the accuracy of the procedures as far as possible, not to determine the gelsemine concentration in all subsequent centesimal dilutions. Perhaps we need to remind readers that normally, when one performs a study of dose-response and the concentration in the highest dose and the dilution factor are known, there is no need to determine the concentration of substances in all successive dilutions.

Another erroneous criticism which forces us to reply is the statement (Chirumbolo, 2014) that our UV-VIS spectra (Marzotto et al., 2014) “showed a peak at 250 nm caused by contaminating millimolar ethanol in *Gelsemium* 2c.” This is untrue, and we fail to understand how the writer could have reached such a conclusion, since UV-visible absorption spectra were performed with a double-beam spectrophotometer using drug samples and reference controls, having exactly the same ethanol concentration. Subsequently the commentary claims that “the authors tested a complex mixture of *G. sempervirens* extract, containing at least about 0.154 mM EtOH at 2c, if dilutions were conducted exactly” and that “concentration of EtOH, set at 30% v/v, faded out to 0.003% in tested dilutions but the authors did not clarify how much for each centesimal dilution in the Methods section.” The writer supposes that high ethanol concentration could have caused “latent apoptosis.” This is confusing and misleading. As clearly indicated in our paper (Marzotto et al., 2014), the final ethanol concentration was 0.03% v/v (p. 2), and “no significant differences in cell viability were observed between cells treated with the ethanol control solution 0.03% (v/v) and untreated cells” (p. 5). Furthermore, as other researchers have found (Do et al., 2013), cell viability of neurocytes is unaffected by doses of ethanol up to 10 mmol/L (0.06%). Low doses of ethanol may influence gene expression, but would have acted in the same way in the *Gelsemium* and in the control samples. To rule out any misinterpretation of our findings, Figure 1 gives a brief outline of our microarray experiments.

**Figure 1:**
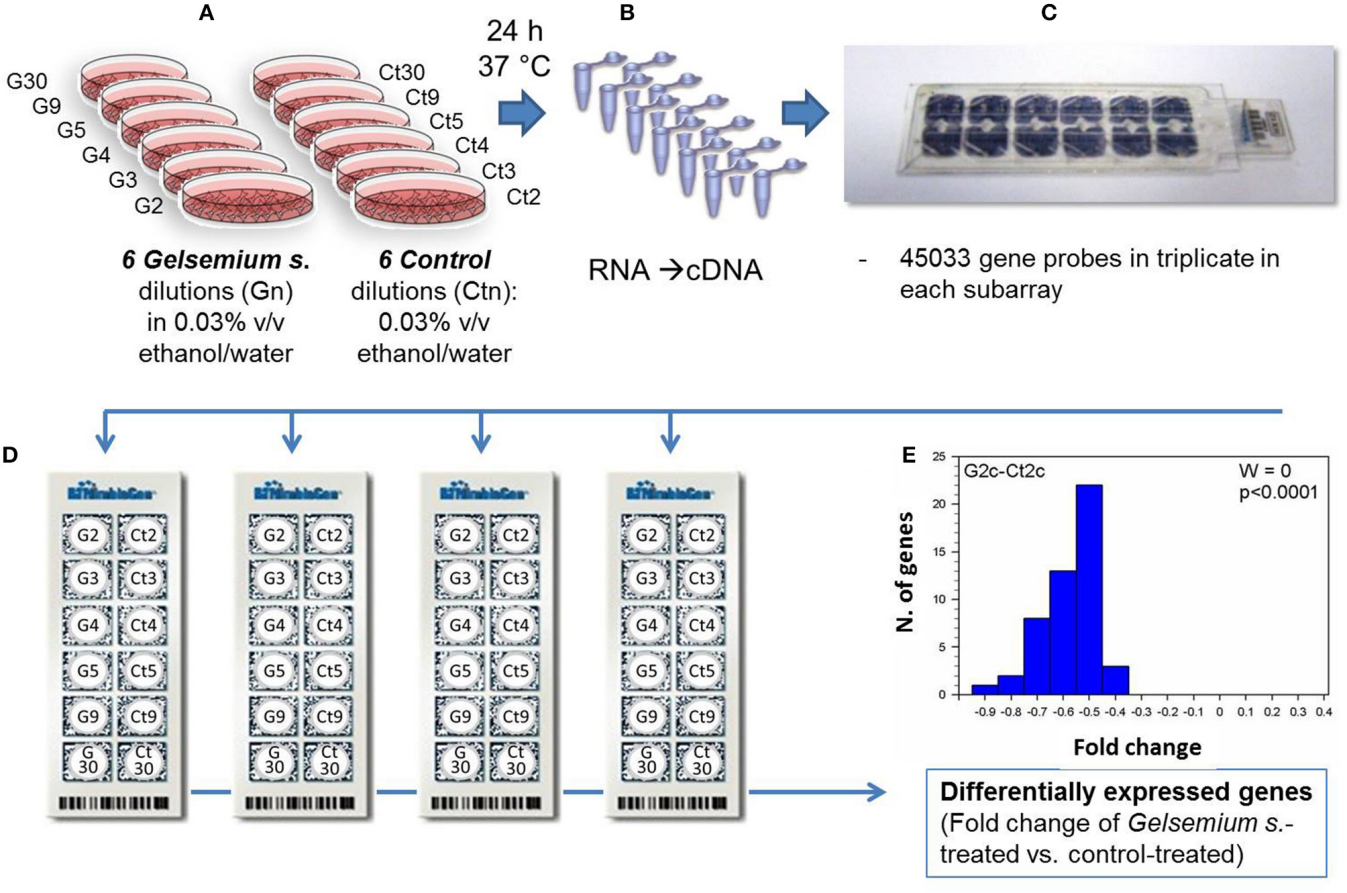
**Workflow of the main steps of the DNA-microarray experiments aimed to discover differentially expressed genes in Gelsemium-treated neuronal cells compared with control treated cells**. Cultured SHSY5Y cells were incubated for 24 h with the Gelsemium dilutions /dynamizations or the corresponding control, both treated with the same ethanol final concentration of 0.03% v/v **(A)**. After the treatment the RNA content was extracted from the cells, cDNA synthesized **(B)** and hybridized on Human Expression microarray **(C)**. In every subarray of the chip, each transcript was targeted with three separate probes, merging the fluorescence values and attributing a statistical score. Four independent replicate experiments were conducted **(D)**. Bioinformatic and statistical analysis of microarray data provided the final results **(E)**. Differentially expressed genes were identified on the 45033 cases using Limma test adjusted for the False Discovery Rate cases using the Benjamini and Hochberg method. Fold change was calculated as Log2-transformed fluorescence value of *Gelsemium* dilutions minus Log2-transformed fluorescence value of the mean of controls. Various *Gelsemium* dilutions were compared with their respective controls using the Friedman test followed by Fisher's exact test. All cultures and tests were performed in sterile conditions using apyrogenic materials and solutions.

Finally, the commentary (Chirumbolo, 2014) maintains that “Gene array profile of expression following 24 h incubation with *Gelsemium* 2c, showed down-regulation of 49 genes, namely 87.5% gene array.” It is hard to understand how this 87.5% of genes was calculated, since the 49 genes represent about 0.1% of the genes on the microarray chip. The observation that “many gene products, listed in the expression profile of 56 genes array, such as LOC154872, KIAA0825, LOC150763, C1orf167, have not been identified” is bizarre. The presence of some as yet unidentified sequences in the human genome database is of course well known.

We hope that these clarifications will be welcomed in the interests of providing correct and truthful information to readers. In summary, we provided reliable evidence that *Gelsemium* exerts a prevalently inhibitory effect on a series of neurocyte genes across a wide dose range (Marzotto et al., 2014; Olioso et al., 2014). The effect decreases with decreasing doses, but whole genome expression analysis made it possible to detect statistically significant changes even at extremely low doses and homeopathic dilutions (e.g., 5th and 9th centesimal dilution, corresponding to final gelsemine concentrations of 6.5 × 10^−15^ mol/L and 6.5 × 10^−23^ mol/L respectively). More robust conclusions about the role of the genes involved will require determination whether proteins encoded by the affected genes are similarly changed, using proteomic and phosphoproteomic approaches, and/or further studies using purified active plant compounds.

## Conflict of interest statement

The authors declare that the research was conducted in the absence of any commercial or financial relationships that could be construed as a potential conflict of interest.
